# Metabolic Dysfunction-Associated Steatotic Liver Disease Is Associated with Increased Risk of Kidney Cancer: A Nationwide Study

**DOI:** 10.3390/cancers16183161

**Published:** 2024-09-15

**Authors:** Juyeon Oh, Beom Kyung Kim, Jin-Ha Yoon, Hyung Ho Lee, Heejoo Park, Jian Lee, Youngsun Park, Byungyoon Yun, Jinsoo Chung

**Affiliations:** 1 Department of Public Health, Graduate School, Yonsei University, Seoul 03722, Republic of Korea; jyoh@yuhs.ac (J.O.); hjpark106@yuhs.ac (H.P.); leejian83@yuhs.ac (J.L.); youngsun0611@yuhs.ac (Y.P.); 2 Department of Internal Medicine, Yonsei University College of Medicine, Seoul 03722, Republic of Korea; beomkkim@yuhs.ac; 3 Institute of Gastroenterology, Yonsei University College of Medicine, Seoul 03722, Republic of Korea; 4 Yonsei Liver Center, Severance Hospital, Yonsei University Health System, Seoul 03722, Republic of Korea; 5 Department of Preventive Medicine, Yonsei University College of Medicine, Seoul 03722, Republic of Korea; flyinyou@gmail.com; 6 The Institute for Occupational Health, Yonsei University College of Medicine, Seoul 03722, Republic of Korea; 7 Institute for Innovation in Digital Healthcare, Yonsei University Health System, Seoul 03722, Republic of Korea; 8 Department of Urology, National Cancer Center, Goyang 10408, Republic of Korea; uroh@ncc.re.kr

**Keywords:** kidney cancer, MASLD, nationwide study, cancer epidemiology

## Abstract

**Simple Summary:**

This study examined the link between metabolic dysfunction-associated steatotic liver disease (MASLD) and kidney cancer risk. Over 8.8 million participants (aged 20–79) were followed for a median of 13.3 years. The study found that participants with MASLD and those with MASLD plus increased alcohol intake (MetALD) had a significantly higher risk of developing kidney cancer compared to those without MASLD. The risk was especially elevated in younger patients. A cumulative relationship between metabolic dysfunction and kidney cancer risk was also observed. The findings highlight the need for a comprehensive approach to metabolic health, particularly focusing on younger individuals.

**Abstract:**

**Background**: This study investigated the association between metabolic dysfunction-associated steatotic liver disease (MASLD) and Kidney Cancer Risk, as the incidence of both diseases gradually increases owing to metabolic health issues. **Methods**: Participants (aged 20–79) undergoing a national health examination between 2009 and 2010 were monitored for new-onset kidney cancer. The MASLD spectrum was classified as non-MASLD, MASLD, or MASLD with increased alcohol uptake (MetALD). Kidney Cancer Risk associated with the MASLD spectrum was estimated using multivariate Cox proportional hazard models. Age- and sex-stratified analyses were also performed. **Results**: Among 8,829,510 participants (median follow-up 13.3 years), the proportion of non-MASLD, MASLD, and MetALD was 64.9%, 30.3%, and 4.7%, respectively, with newly developed kidney cancer in 17,555 participants. Kidney cancer was significantly increased with MASLD (adjusted hazard ratio [aHR] 1.51, 95% confidence interval [CI] 1.46–1.56) and MetALD (aHR 1.51, 95% CI 1.42–1.61), compared with the non-MASLD group. Kidney Cancer Risk was the highest among young populations (aHR 1.93, 95% CI 1.77–2.11 for MASLD and aHR 1.91, 95% CI 1.65–2.22 for MetALD), according to stratification analysis. Furthermore, the cumulative relationship between metabolic dysfunction and Kidney Cancer Risk was confirmed across all MASLD spectra. **Conclusions**: Our study highlights the positive association between MASLD and Kidney Cancer Risk, emphasizing a comprehensive approach to metabolic health. This also serves as a call to devote closer attention to the metabolic health of younger patients.

## 1. Introduction

The global incidence of kidney cancer has been increasing, making this disease a significant oncological concern [1]. Kidney cancer is typically diagnosed at an advanced stage, leading to limited treatment options and a poor prognosis. Furthermore, it is partly attributed to the increasing prevalence of metabolic syndrome influenced by Westernized dietary habits and lifestyles [2]. Previous studies have identified components of metabolic dysfunction, such as increased waist circumference, triglycerides, fasting blood glucose, and blood pressure, as risk and prognostic factors for kidney cancer [3,4]. Such findings prompt the need for further studies to identify high-risk patients for effective screening and appropriate treatment.

Hepatic steatosis has been increasingly recognized as a critical global metabolic health concern as the prevalence of metabolic syndrome and/or obesity simultaneously increases [5,6,7]. The terminology for hepatic steatosis has undergone significant changes. Initially defined as non-alcoholic fatty liver disease (NAFLD), this classification failed to address metabolic factors, alcohol consumption, and other etiologies, complicating risk stratification [8,9,10,11]. Later, metabolic dysfunction-associated fatty liver disease (MAFLD) was introduced to include metabolically complex liver conditions that overlapped with chronic liver diseases of various etiologies not covered by NAFLD [1,12,13]. However, MAFLD could not account for mixed etiologies involving both metabolic factors and alcohol consumption, nor could it include lean patients with hepatic steatosis [14]. Consequently, a new classification system, steatotic liver disease (SLD), was introduced, recognizing the interaction between metabolic conditions and alcohol consumption. This led to the development of new terms: metabolic dysfunction-associated steatotic liver disease (MASLD) and MASLD with increased alcohol consumption (MetALD) [15,16,17,18].

Previous studies assessing the effects of metabolic components on kidney outcomes have primarily focused on chronic kidney disease (CKD) rather than kidney cancer [19,20,21,22]. Moreover, although various studies have investigated the association between hepatic steatosis and extrahepatic malignancies, few have assessed its association with kidney cancer [23,24,25,26].

Hence, this study aimed to evaluate the association between MASLD and the risk of kidney cancer by incorporating demographic stratification to ascertain the effects of metabolic dysfunction on the risk of kidney cancer.

## 2. Materials and Methods

This nationwide cohort study used data from the National Health Insurance Service (NHIS) database in the Republic of Korea, which contains information on 97.2% of the entire population [27]. The database contains an array of demographic and socioeconomic characteristics, information on outpatient visits or hospitalizations, diagnostic codes, health checkup data, and comprehensive drug prescriptions. The NHIS conducts a comprehensive biennial health examination for adults. This examination encompasses clinical and biochemical tests, along with the collection of lifestyle information through structured questionnaires. Study subjects ranging in age from 20 to 79 years who underwent a national health examination between 2009 and 2010 were included. The date of the health examination was designated as the index date for each participant.

The study implemented specific exclusion criteria as follows: incomplete data regarding residential area or household income, or lacking values in measurements or blood tests; history of concurrent liver disease including viral hepatitis (B15–B19), alcoholic liver disease (K70), or documented alcohol intake exceeding 420 g/week for males or 350 g/week for females, toxic liver disease (K71), biliary cholangitis (K74.3–K74.5), autoimmune hepatitis (K75.4), Wilson’s disease (E83.0), and hemochromatosis (E83.1); and history of any type of malignancy.

This study adhered to the ethical principles of the Declaration of Helsinki and Istanbul and was approved by the Institutional Review Board of Severance Hospital (IRB number: 4-2022-0813). The requirement for informed consent was waived owing to the retrospective nature of this study.

### 2.1. Main Outcomes

The primary outcome of this study was the incidence of new-onset kidney cancer, and all-cause mortality was considered the secondary outcome. The diagnosis of kidney cancer was based on the first recorded hospital encounter, coded as C64 in the International Classification of Diseases, 10th Revision (ICD-10), along with code V193. These data were derived from a registry initiative implemented by the government of the Republic of Korea in 2006, aimed at reducing copayments for rare and challenging diseases [28]. The follow-up period for the patients was extended until the development of kidney cancer, death, or December 2022, whichever occurred first.

### 2.2. Variables and Covariates

The MASLD classification in this study was based on the simultaneous presence of SLD and ≥1 cardiometabolic risk factors. As such, MASLD was characterized by a fatty liver index (FLI) ≥ 30, a criterion based on and aligned with methodologies used in other Asian research studies [29]. Cardiometabolic risk factors encompassed the following: body mass index (BMI) ≥ 23 kg/m^2^ or a waist circumference (WC) ≥ 90 cm for males and ≥80 cm for females; fasting glucose level ≥ 100 mg/dL, diagnosis of type 2 diabetes, or the use of glucose-lowering medications; blood pressure ≥ 130/85 mmHg or use of antihypertensive drugs; triglyceride concentrations ≥ 150 mg/dL or use of lipid-lowering medications; or low high-density lipoprotein cholesterol levels, defined as <40 mg/dL for males and <50 mg/dL for females, or use of lipid-lowering drugs. Among individuals diagnosed with MASLD, those who reported moderate alcohol consumption levels (weekly intake, 210–420 g for males and 140–350 g for females, in which 1 “shot” is equivalent to 10 g of alcohol) were classified as MetALD—more specifically, MASLD and increased alcohol consumption. Consequently, the individuals were primarily categorized into three groups: non-MASLD, MASLD, and MetALD.

The covariates integrated into the analysis included age, sex, residential area (capital, metropolitan, or other), household income quartile, employment status, smoking history, physical activity, and Charlson Comorbidity Index (CCI). Employment status was determined on the basis of the insurance type reported in the NHIS database for the index year. Based on lifestyle questionnaires, smoking status was categorized into non-smokers, former smokers, and current smokers. Physical activity levels were assessed by calculating the metabolic equivalent of tasks (METs)-h/week, summing the total vigorous (7 METs), moderate (4 METs), and walking (2.9 METs) activities reported [30]. Physical activity was subdivided into four categories according to METs-min/week: 0–499, 500–999, 1000–1499, and ≥1500. The updated CCI was calculated considering diagnostic codes for each disease category, based on ≥1 hospitalization(s) or ≥3 outpatient visits before the index date [31]. CKD was defined as an estimated glomerular filtration rate (eGFR) < 60 mL/min/1.73 m^2^, derived from national health examination results.

### 2.3. Statistical Analysis

Initial participant characteristics are expressed as a median (interquartile range [IQR]) or proportion (number). The cumulative incidence rates of kidney cancer and all-cause mortality in each group were calculated using the Kaplan–Meier method and compared using the log-rank test. This study applied multivariable Cox proportional hazard models to determine the adjusted hazard ratios (HRs) and corresponding 95% confidence intervals (CIs) for both primary and secondary outcomes. Model 1 included age and sex; Model 2 incorporated additional socioeconomic variables such as residential area, household income, and economic activity; and Model 3 included variables such as smoking history, physical activity, CKD, and CCI based on Model 2.

Multiple sensitivity analyses were also conducted. Stratified analyses were performed to determine kidney cancer risk according to age, sex, and the use of type 2 diabetes and statin medications. Age was grouped into three categories in the stratified analysis: 20–39, 40–64, and 65–79 years. Patients with a CCI score ≥ 6 were excluded. In addition, the primary analysis was replicated using different biochemical SLD models: FLI ≥ 60 (cut-off for severe SLD) [32]; FLI ≥ 31 for males and ≥18 for females [33]; and hepatic steatosis index (HSI) ≥ 36 [34].

Moreover, the cumulative relationship between the number of metabolic dysfunction components (ranging from 0 [none present] to 5 [all present]) and kidney cancer was investigated and summarized using heatmaps. This approach enabled statistical assessment of the cumulative effects of metabolic dysfunction on health risks. The risk for kidney cancer associated with the number of metabolic dysfunctions was also assessed with subgroups with FLI 30–59, or ≥60, and alcohol consumption level (either MASLD or MetALD).

All statistical analyses were performed using SAS Enterprise version 7.1 (SAS Institute, Cary, NC, USA) and R version 4.0.3 (R Foundation for Statistical Computing, Vienna, Austria). All estimates were two-sided, and differences were considered statistically significant at *p* < 0.05.

## 3. Results

### 3.1. Baseline Characteristics of Patients among the Entire Population

**Figure 1:**
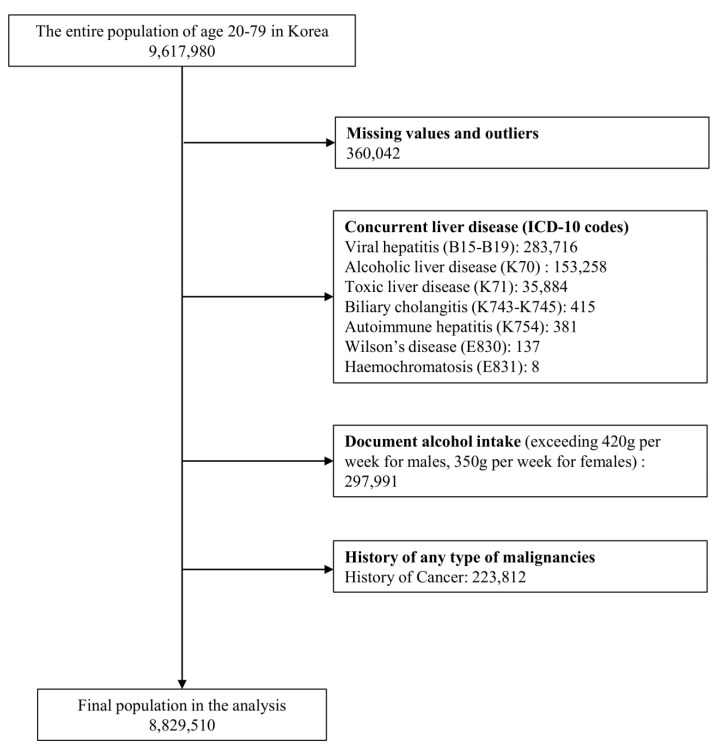
Flow chart of the participant inclusion process.

Among the initial cohort of 9,617,980 patients, the final analysis included data from 8,829,510 individuals after excluding specific cases (Figure 1). The baseline characteristics of the patients are summarized in Table 1. Males accounted for 54.1% of the entire population, with a median age of 46.53 years (IQR: 36–56 years). Overall, 64.9% (*n* = 5,731,764) of the population did not have MALD, 30.3% (*n* = 2,679,407) had MASLD, and 4.7% (*n* = 418,339) had MetALD. An incremental increase was observed from non-MASLD to MASLD and then to MetALD groups in terms of the proportion of participants 40–59 years of age, male sex, and those engaged in economic activities (*p* < 0.001). Additionally, individuals in the MASLD and MetALD groups indicated a lower proportion of chronic kidney disease compared to the non-MASLD group (*p* < 0.001). Relative to the non-MASLD group, individuals in both the MASLD and MetALD groups had a higher proportion of above-average income, residing in non-metropolitan areas, CCI scores ≥ 2, smoking history, and <500 METs-min/week of physical activity (*p* < 0.001).

### 3.2. Cumulative Risk for Primary and Secondary Outcomes According to MASLD or MetALD Compared with Non-MASLD

**Figure 2:**
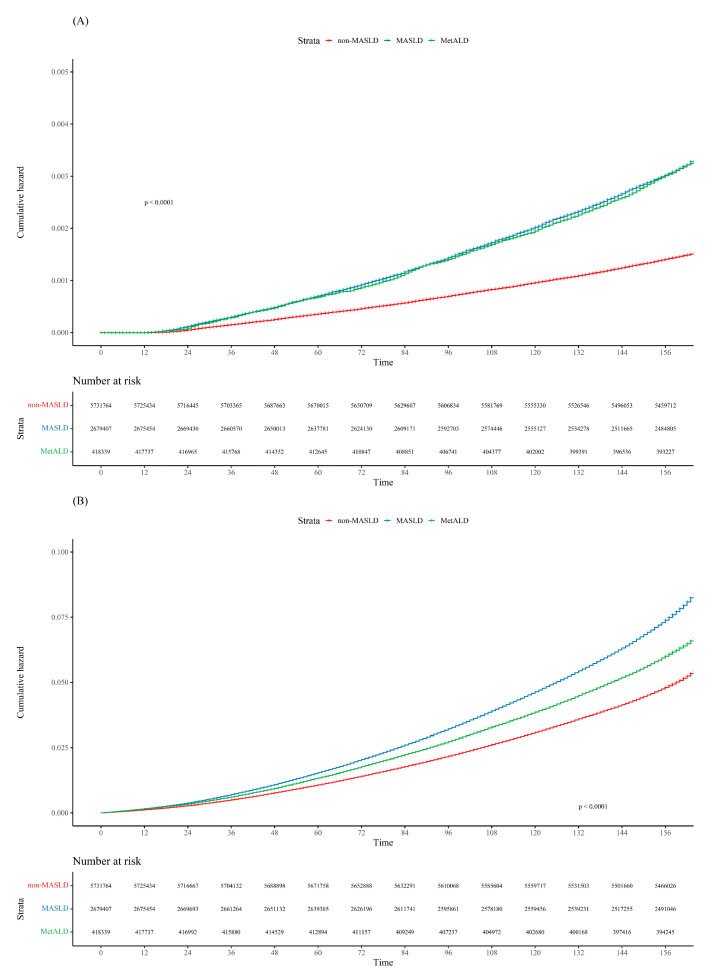
Cumulative incidence plot of (**A**) kidney cancer and (**B**) all-cause mortality according to the MASLD spectrum.

Over a median follow-up period of 13.25 years, 17,555 participants (0.20% of the total) developed kidney cancer. Among them, 8179 (0.15%), 8105 (0.19%), and 822 (0.009%) patients were classified into the non-MASLD, MASLD, and MetALD groups, respectively. The age-standardized 5-year cumulative incidence rates of kidney cancer were 0.04% for the non-MASLD group and 0.07% for both the MASLD and MetALD groups. Statistical analysis showed significant differences, with *p* < 0.001 between the non-MASLD vs. MASLD groups and non-MASLD vs. MetALD groups, while there was no significant difference between MASLD and MetALD (*p* = 0.97) (Figure 2A).

During the follow-up period, 539,834 patients died. The all-cause mortality rates in the non-MASLD, MASLD, and MetALD groups were 5.09%, 5.73%, and 5.80%, respectively (*p* < 0.001). The age-standardized 5-year cumulative incidence rates of all-cause mortality were 1.07% in the non-MASLD group, 1.53% in the MASLD group, and 1.33% in the MetALD group. Statistical analysis revealed significant differences among the groups, with *p*-values of <0.001 for non-MASLD vs. MASLD, non-MASLD vs. MetALD, and MASLD vs. MetALD (Figure 2B).

The data reported in Table 2 show that both MASLD and MetALD were significantly associated with a higher risk of kidney cancer through Model 3 using Cox proportional hazard models: adjusted HR 1.51 (95% CI, 1.46–1.56) for the MASLD group and 1.51 (95% CI, 1.42–1.61) for the MetALD group. The analysis indicated a similar risk of kidney cancer between the MASLD and MetALD groups (*p* = 0.97).

Similarly, both MASLD and MetALD, compared with non-MASLD, were significantly associated with higher all-cause mortality: adjusted HR 1.09 (95% CI, 1.09–1.11) for the MASLD group and 1.19 (95% CI, 1.17–1.20) for the MetALD group. In contrast, the MetALD group exhibited a slightly higher all-cause mortality than the MASLD group.

### 3.3. Stratification Analyses of the Risk for Kidney Cancer According to MASLD or MetALD Compared with Non-MASLD

Stratification analyses according to age and sex for the risk of kidney cancer according to MASLD or MetALD compared with non-MASLD are summarized in Table 3. The risk of kidney cancer in the MASLD and MetALD groups remained significantly higher than that in the non-MASLD group, consistently among all age and sex subgroups (*p* < 0.001). Furthermore, similar to the main analyses, a comparable risk of kidney cancer between the MASLD and MetALD groups was observed in all age and sex subgroups (*p* > 0.001).

Notably, the impact of both MASLD and MetALD compared with non-MASLD for the risk for kidney cancer was the most prominent among the young age subgroup than among the middle and older subgroups (adjusted HR 1.91–1.93 vs. 1.32–1.51, respectively). In the stratification analyses based on the use of type 2 diabetes and statin medications (Table S1), the results indicated that individuals not taking diabetes or statin medications had a slightly higher risk of kidney cancer associated with MASLD/MetALD compared to those without MASLD.

Results of the sensitivity analysis, which included patients after excluding those with a CCI score ≥ 6 and those with various cut-off values to define SLD, are summarized in Table S2. Similar results were consistently reproduced; both the MASLD and MetALD groups exhibited a higher risk of kidney cancer than the non-MASLD group, and there was no significant difference between the MASLD and MetALD groups.

### 3.4. Analysis of the Risk for Kidney Cancer in Relation to Metabolic Burden Defined as the Number of Metabolic Components

**Figure 3:**
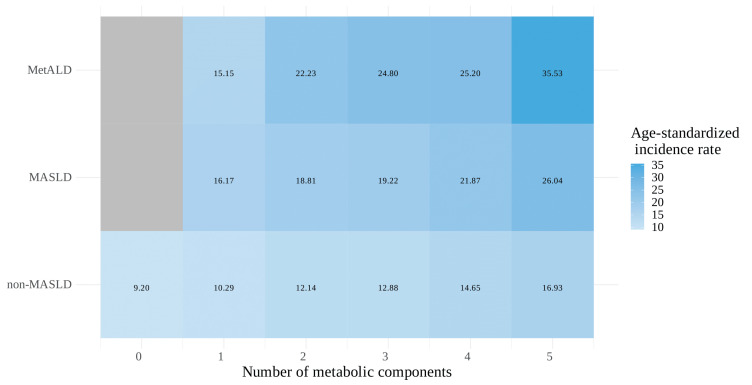
Heatmap of the age-standardized incidence rate (per 100,000 person-years) for kidney cancer associated with the number of metabolic components across the MASLD spectrum.

The correlation between kidney cancer risk and metabolic burden, defined as the number of metabolic components (ranging from 0 [none present] to 5 [all present]), was further assessed across the MASLD spectrum (the number of metabolic components starting from 1 to 5 in the MASLD and MetALD groups). A cumulative relationship between the risk of kidney cancer and the number of metabolic components was observed in all MASLD categories, more specifically, non-MASLD, MASLD, and MetALD (Table 4). This relationship is also depicted in a heatmap illustrating the age-standardized incidence rate of kidney cancer (Figure 3).

In additional sensitivity analyses, the cumulative relationship between the risk of kidney cancer and the number of metabolic components persisted among various subgroups defined using alcohol consumption and/or different FLI cut-off values (Figure S1).

## 4. Discussion

In this comprehensive study based on a nationwide representative cohort in the Republic of Korea, we investigated the association between kidney cancer risk and MASLD or MetALD. We found that both MASLD and MetALD were significantly associated with an increased risk of kidney cancer compared with non-MASLD. The effect of increased alcohol intake within 420 g/week for males and 350 g/week for females, in addition to MASLD (the so-called MetALD), was negligible in increasing the risk of kidney cancer. Similar trends were observed in stratification analyses according to age and sex. To date, research investigating the risk factors of kidney cancer has been constrained by the relatively low incidence of the disease, which limits the statistical power to detect associations. However, primarily because the incidence of kidney cancer has been gradually increasing (to 431,288 new cases in 2020) [2], the need for comprehensive research investigating the risk factors for kidney cancer has become an important health issue.

Our study has several strengths. Using a large-scale nationwide dataset from the Republic of Korea, we drew robust conclusions regarding the positive association between kidney cancer risk and MASLD or MetALD. Approximately 25% of kidney cancers can be attributed to overweight or obesity [35]. Our study underscores the importance of controlling other metabolic dysfunctions such as hepatic steatosis, dyslipidemia, insulin resistance, and hypertension, in addition to obesity, for the primary prevention of kidney cancer. Second, by analyzing different age groups, we highlighted that the detrimental effect of metabolically unhealthy conditions on the risk for kidney cancer was the most prominent in the young age group compared with the middle and older subgroups (adjusted HR 1.91–1.93 vs. 1.32–1.51, respectively). This is a noteworthy finding, given that the incidence of kidney cancer among younger patients has steeply increased for several decades, possibly resulting from shifts in dietary habits and an increase in metabolic dysfunction [36,37,38]. Although the overall incidence of kidney cancer in the older patients remains higher than that in the young(er) patients, the carcinogenic effect of metabolic dysfunction is strongest in the young age group. This could be attributed to the generally lower baseline risk for kidney cancer in younger age groups, making the relative increase in the detrimental effects of metabolic dysfunction more pronounced. According to the baseline characteristics of metabolic components and lifestyle factors in the participation from each age group (Table S3), younger patients (age < 40) showed the biggest difference in BMI, the proportion of current smokers, blood pressure, and fasting blood sugar level between non-MASLD and MASLD/MetALD populations. Moreover, several murine studies specifically show that obesity and obesogenic diets not only raise the likelihood of developing malignancies but also speed up their progression and lead to their onset at younger ages [36,39,40]. This also emphasizes that younger patients are more susceptible to the oncogenic effects of metabolic dysfunction, indicating the importance of controlling metabolic dysfunction for effective prevention in specific populations. Finally, we confirmed consistent results using various stratification and sensitivity analyses.

However, the pathogenesis underlying this association remains unclear. The most likely explanation is that the accumulation and dysfunction of excess adipose tissue create an ideal environment for the initiation and progression of kidney cancer. This may be linked to factors such as hormonal imbalances, chronic tissue hypoxia, heightened inflammation, altered cellular energy metabolism, increased angiogenesis, epithelial-to-mesenchymal transition, and genomic instability. These changes connect unhealthy metabolic conditions with an increased risk of kidney cancer [41]. Interleukin-6 (IL-6), a significant marker in the pathogenesis of metabolic syndrome and its cardiovascular complications [42], also induces 5′ AMP-activated protein kinase phosphorylation, a critical process for IL-6-mediated glucose uptake and lipid oxidation [43], which is implicated in obesity-related cancers [44]. Moreover, a recent review highlighted visceral obesity’s involvement in kidney cancer, further emphasizing the metabolic impact on tumorigenesis [45]. In obesity, the expansion of adipose tissue leads to hypoxia, which triggers compensatory mechanisms like angiogenesis to restore oxygen supply. Hypoxia activates hypoxia-inducible factors (HIFs), specifically, HIF-1α and HIF-2α, which regulate several pathways related to metabolism, angiogenesis, and tumor growth [46]. In addition, the role of adipokines, such as leptin and adiponectin, in modulating inflammatory responses and influencing cancer progression has gained attention [47]. Leptin, often elevated in obese individuals, promotes cell proliferation and angiogenesis [48], while adiponectin has been linked to increased antitumor properties through suppressing mTOR and Stat3 pathways and stimulating the activity of 5′AMP-activated protein kinase (AMPK) [49]. These adipokines, in conjunction with HIF-mediated responses, further create a microenvironment conducive to tumor growth. Understanding the interplay among these factors may reveal novel therapeutic targets for obesity-associated kidney cancer. Further studies investigating the biological and epidemiological aspects are required to address these issues.

To address this unmet need, we assessed the correlation between the number of metabolic dysfunctions and the risk of kidney cancer and demonstrated that the risk increased incrementally with the aggregation of metabolic risk factors. Such a cumulative relationship shown in the heatmaps was reproduced not only in the main analyses but also in the sensitivity analyses according to FLI cut-offs and/or alcohol intake. Conversely, those with higher FLI (≥60) consistently exhibited an overall higher risk for kidney cancer than those with lower FLI (30–59) at every stratum according to the number of metabolic components, suggesting that significant intrahepatic fat accumulation may be regarded as another component defining the so-called “metabolic syndrome” in addition to the five existing criteria. Indeed, numerous studies have demonstrated a strong link between liver triacylglycerol accumulation—specifically, when exceeding 55 mg/g liver (5.5%), a threshold indicative of NAFLD—and visceral adipose tissue and insulin resistance, suggesting that liver triacylglycerol plays a crucial role in metabolic dysregulation [50,51,52].

The present study has several limitations. First, we defined SLD based on biochemical markers rather than imaging or histopathology, potentially leading to misclassification. However, to overcome this drawback, we performed sensitivity analyses using various biochemical scoring cutoffs, striving to ensure the accuracy of the SLD classification despite the absence of imaging or histopathological data. Second, another issue is that the NHIS database lacks data regarding specific variables such as dietary habits and genetic factors, which could act as unmeasured confounders, potentially introducing bias into our results. Third, different classes of therapies may have varying effects on the disease course of MASLD [53]. While data on therapies influencing metabolic status, such as diabetes or lipid-lowering medications, were available, their potential impact on the association between liver steatotic disease and kidney cancer was not fully explored in this study. Further analysis is needed to understand how these treatments may have influenced the findings. Finally, because this study focused on the population of the Republic of Korea, the findings may not apply to other ethnic or racial groups. Further studies are required to confirm these findings, as the impact of MASLD on kidney cancer may vary by race or ethnicity [54].

## 5. Conclusions

Our study delineates a positive association between MASLD and the risk of kidney cancer, emphasizing the importance of a comprehensive approach to metabolic health. As the demographic profile of kidney cancer has shifted toward younger ages, our findings emphasize the urgent need for preventive strategies to address the multifactorial nature of this disease.

## Figures and Tables

**Table 1:** Baseline characteristics of participants according to MASLD spectrum.

	Non-MASLD (*n* = 5,731,764)	MASLD (*n* = 2,679,407)	MetALD (*n* = 418,339)
Age			
20–39	2,023,106 (35.3)	695,594 (25.96)	136,490 (32.63)
40–59	3,084,660 (53.82)	1,581,351 (59.02)	257,568 (61.57)
60–79	623,998 (10.89)	402,462 (15.02)	24,281 (5.8)
Sex			
Male	2,364,378 (41.25)	1,912,048 (71.36)	391,332 (93.54)
Female	3,367,386 (58.75)	767,359 (28.64)	27,007 (6.46)
Type of Insurance			
Employee	3,775,951 (65.88)	1,884,263 (70.32)	353,519 (84.51)
Self-Employed	1,955,813 (34.12)	795,144 (29.68)	64,820 (15.49)
Income Quartile			
(Premium Insurance, KRW)			
High	1,349,674 (23.55)	753,276 (28.11)	111,234 (26.59)
High-middle	1,357,747 (23.69)	725,883 (27.09)	119,631 (28.6)
Low-middle	1,518,673 (26.5)	627,702 (23.43)	107,316 (25.65)
Low	1,505,670 (26.27)	572,546 (21.37)	80,158 (19.16)
Residential Area			
Capital	1,119,023 (19.52)	495,329 (18.49)	77,751 (18.59)
Metropolitan	1,547,452 (27)	704,732 (26.3)	110,878 (26.5)
Others	3,065,289 (53.48)	1,479,346 (55.21)	229,710 (54.91)
Chronic Kidney Disease			
No	4,959,732 (86.53)	2,451,292 (91.49)	404,299 (96.64)
Yes	772,032 (13.47)	228,115 (8.51)	14,040 (3.36)
Charlson Comorbidity Index			
0	4,449,249 (77.62)	1,850,746 (69.07)	323,297 (77.28)
1	771,468 (13.46)	381,406 (14.23)	39,661 (9.48)
≥2	511,047 (8.92)	447,255 (16.69)	55,381 (13.24)
Smoking History			
non-smoker	3,998,494 (69.76)	1,312,370 (48.98)	77,234 (18.46)
ex-smoker	606,348 (10.58)	501,184 (18.71)	104,369 (24.95)
current smoker	1,126,922 (19.66)	865,853 (32.32)	236,736 (56.59)
Physical Activity			
<500	279,270 (4.87)	132,476 (4.94)	22,899 (5.47)
500 to <1000	574,254 (10.02)	274,127 (10.23)	49,031 (11.72)
1000 to <1500	1,584,680 (27.65)	733,660 (27.38)	125,009 (29.88)
≥1500	3,293,560 (57.46)	1,539,144 (57.44)	221,400 (52.92)

Abbreviations: MASLD, metabolic dysfunction-associated steatotic liver disease; ALD, alcohol-associated liver disease.

**Table 2:** Adjusted HR (95% CI) of KC associated with metabolic SLDs.

Outcome	Metabolic SLDs	Crude Model	Model 1	Model 2	Final Model
KC	Non-MASLD	1.00 (reference)	1.00 (reference)	1.00 (reference)	1.00 (reference)
	MASLD	2.15 (2.08–2.21)	1.55 (1.50–1.60)	1.55 (1.50–1.60)	1.51 (1.46–1.56)
	MetALD	2.15 (2.03–2.28)	1.57 (1.48–1.67)	1.57 (1.48–1.67)	1.51 (1.42–1.61)
All-cause mortality	Non-MASLD	1.00 (reference)	1.00 (reference)	1.00 (reference)	1.00 (reference)
	MASLD	1.54 (1.53–1.55)	1.07 (1.06–1.08)	1.06 (1.07–1.08)	1.09 (1.09–1.10)
	MetALD	1.24 (1.23–1.26)	1.24 (1.22–1.25)	1.24 (1.22–1.25)	1.19 (1.17–1.20)

Model 1 was adjusted for age and sex. Model 2 was adjusted for age, sex, residential area, household income, and economic activity. Model 3 was adjusted for age, sex, residential area, household income, economic activity, smoking history, physical activity, CKD, and CCI. Abbreviations: SLD, steatotic liver disease; MASLD, metabolic dysfunction-associated steatotic liver disease; ALD, alcohol-associated liver disease; CKD, chronic kidney disease; CCI, Charlson Comorbidity Index; KC, kidney cancer.

**Table 3:** Stratified analyses on the association of KC risk with metabolic SLD by age and sex.

	Metabolic SLDs	N at Event	Person-Year	Rate (Per 100,000)	Adjusted HR (95% CI)
Age					
Young (20–39)	Non-MASLD	1206	26,786,386	4.5	Reference (1.00)
	MASLD	1154	9,183,713	12.57	1.93 (1.77–2.11)
	MetALD	224	1,798,528	12.45	1.91 (1.65–2.22)
Middle (40–59)	Non-MASLD	5102	40,612,225	12.56	Reference (1.00)
	MASLD	5248	20,653,918	25.41	1.51 (1.45–1.55)
	MetALD	879	3,345,501	26.27	1.45 (1.34–1.56)
Elder (60–79)	Non-MASLD	1871	7,481,997	25.01	Reference (1.00)
	MASLD	1703	4,828,358	35.27	1.32 (1.24–1.41)
	MetALD	168	283,212	59.32	1.50 (1.28–1.77)
Sex					
Male	Non-MASLD	4662	30,620,323	15.23	Reference (1.00)
	MASLD	6447	24,727,118	26.07	1.58 (1.52–1.64)
	MetALD	1232	5,073,089	24.29	1.57 (1.47–1.67)
Female	Non-MASLD	3517	44,260,285	7.95	Reference (1.00)
	MASLD	1658	9,938,871	16.68	1.42 (1.33–1.51)
	MetALD	39	354,152	11.01	1.38 (1.00–1.90)

All models were adjusted for age, sex, residential area, household income, economic activity, smoking history, physical activity, CKD, and CCI. Abbreviations: KC, kidney cancer; SLD, steatotic liver disease; MASLD, metabolic dysfunction-associated steatotic liver disease; MetALD, MASLD with increased alcohol intake; ALD, alcohol-associated liver disease; CKD, chronic kidney disease; CCI, Charlson Comorbidity Index.

**Table 4:** Risk of KC associated with metabolic SLDs according to the number of metabolic components.

Metabolic SLDs	Number of Metabolic Components	N at Risk	N of KC	Person-Year	Rate (Per 100,000)	Adjusted HR (95% CI)
Non-MASLD	0	1,660,901	1373	21,901,403	6.269	reference (1.00)
	1	1,825,417	2215	23,927,946	9.257	1.14 (1.07–1.22)
	2	1,273,925	2236	16,578,036	13.488	1.38 (1.29–1.48)
	3	616,847	1317	7,964,314	16.536	1.48 (1.37–1.61)
	4	258,751	723	3,295,460	21.939	1.78 (1.62–1.96)
	5	95,923	315	1,213,450	25.959	1.89 (1.67–2.15)
MASLD	1	251,788	514	3,297,716	15.587	reference (1.00)
	2	653,856	1619	8,528,412	18.984	1.14 (1.03–1.26)
	3	821,516	2321	10,654,397	21.784	1.23 (1.11–1.35)
	4	631,805	2270	8,134,048	27.907	1.48 (1.35–1.64)
	5	320,442	1381	4,051,415	34.087	1.67 (1.50–1.85)
MetALD	1	44,951	85	586,151	14.501	reference (1.00)
	2	113,522	283	1,478,212	19.145	1.24 (0.97–1.58)
	3	137,568	406	1,785,078	22.744	1.36 (1.08–1.72)
	4	91,067	321	1,177,025	27.272	1.51 (1.18–1.92)
	5	31,231	176	400,774	43.915	2.09 (1.60–2.72)

All models were adjusted for age, sex, residential area, household income, economic activity, smoking history, physical activity, CKD, and CCI. Abbreviations: KC, kidney cancer; SLD, steatotic liver disease; MASLD, metabolic dysfunction-associated steatotic liver disease; MetALD, MASLD with increased alcohol intake; ALD, alcohol-associated liver disease; CKD, chronic kidney disease; CCI, Charlson Comorbidity Index.

## Data Availability

Data sharing is not applicable due to the policy of NHIS.

## Supplementary Materials

The following supporting information can be downloaded at: https://www.mdpi.com/article/10.3390/cancers16183161/s1, Table S1. Stratified analyses based on the use of medications regarding the association of KC risk with metabolic SLD; Table S2. Sensitivity analyses on the risk of KC associated with metabolic SLD with varying cut-off and scoring system; Table S3. The proportion of metabolic components and lifestyle factors among participants stratified by age; Figure S1: A heatmap for the risk of kidney cancer associated with the combination of fatty liver disease, alcohol intake, and the number of metabolic components.
