# Supplementary material for: Metabolic Dysfunction-Associated Steatotic Liver Disease Is Associated with Increased Risk of Kidney Cancer: A Nationwide Study
Source: Cancers (Basel). 2024 Sep 15;16(18):3161. doi: 10.3390/cancers16183161 (PMC11430135; doi:10.3390/cancers16183161)
Supplement: Supplementary file 1 [file cancers-16-03161-s001.zip › cancers-3194899-supplementary.pdf]

cancers MDPI

Table S1. Stratified analyses based on the use of medications regarding the association of KC risk with metabolic SLD.

	Metabolic SLDs	N of Event	Person- Years	Rate (Per 100,000 Person-Years)	Adjusted HR (95% CI)
Diabetes medication					
No	Non-MASLD	7644	72,726,563	10.51	Reference (1.00)
	MASLD	7158	31,959,345	22.4	1.51 (1.46–1.57)
	MetALD	1153	5,149,674	22.39	1.51 (1.42–1.61)
Yes	Non-MASLD	535	2,154,045	24.84	Reference (1.00)
	MASLD	947	2,706,644	34.99	1.33 (1.19–1.48)
	MetALD	118	277,567	42.51	1.38 (1.12–1.70)
Statin medication					
No	Non-MASLD	7255	70,648,451	10.27	Reference (1.00)
	MASLD	6479	29,828,254	21.61	1.50 (1.45–1.55)
	MetALD	1073	4,942,796	21.71	1.50 (1.40–1.60)
Yes	Non-MASLD	924	4,232,157	21.86	Reference (1.00)
	MASLD	1658	4,837,735	34.29	1.35 (1.24–1.47)
	MetALD	198	484,445	40.84	1.36 (1.15–1.59)

Table S2. Sensitivity analyses on the risk of KC associated with metabolic SLD with varying cut-off and scoring system.

	Metabolic SLDs		
Sensitivity analyses	Non-MASLD	MASLD	MetALD
excluding individuals with CCI 6 or more	reference (1.00)	1.51 (1.46–1.56)	1.51 (1.42–1.61)
FLI with cut-off value of 60	reference (1.00)	1.58 (1.52–1.64)	1.52 (1.41–1.65)
FLI with cut-off value of 31 for male and 18 for female	reference (1.00)	1.51 (1.47–1.56)	1.52 (1.43–1.61)
HSI with cut-off value of 36	reference (1.00)	1.52 (1.47–1.57)	1.61 (1.47–1.75)

All models were adjusted for age, sex, residential area, household income, economic activity, smoking history, physical activity, CKD, and CCI. Abbreviation: KC, kidney cancer; SLD, steatotic liver disease; MASLD, metabolic dysfunction-associated steatotic liver disease; MetALD, MASLD with increased alcohol intake; ALD, alcohol-associated liver disease; CKD, Chronic kidney disease; CCI, charlson comorbidity index; FLI, fatty liver disease; HSI, hepatic steatosis index.

Table S3. The proportion of metabolic components and lifestyle factors among participants stratified by age.

Age group	<40			40–64			≥65		
**Variable**	**non-MASLD**	**MASLD**	**MetALD**	**non-MASLD**	**MASLD**	**MetALD**	**non-MASLD**	**MASLD**	**MetALD**
Obesity									
No	1445322 (71.44)	42408 (6.10)	12371 (9.06)	1675987 (54.33)	111531 (7.05)	33338 (12.94)	303388 (48.62)	23189 (5.76)	4238 (17.45)
Yes	688895 (28.56)	653186 (93.90)	124119 (90.94)	1408673 (45.67)	1469820 (92.95)	224230 (87.06)	320610 (51.38)	379273 (94.24)	20043 (82.55)
Smoking history									
Non-smoker	1254336 (62.0)	220126 (31.65)	18322 (13.42)	2269832 (73.58)	798754 (50.51)	50375 (19.56)	474326 (76.01)	293490 (72.92)	8537 (35.16)
Ex-smoker	196197 (9.7)	107772 (15.49)	23155 (16.96)	334654 (10.85)	331160 (20.94)	73473 (28.53)	75497 (12.1)	62252 (15.47)	7741 (31.88)
Current-smoker	572573 (28.3)	367696 (52.86)	95013 (69.61)	480174 (15.57)	451437 (28.55)	133720 (51.92)	74175 (11.89)	46720 (11.61)	8003 (32.96)
Physical activity (METs-min/week)									
≥1500	62981 (3.11)	22620 (3.25)	4815 (3.53)	172443 (5.59)	82508 (5.22)	15430 (5.99)	43846 (7.03)	27348 (6.8)	2654 (10.93)
1000–1499	173338 (8.57)	65421 (9.41)	14174 (10.38)	340864 (11.05)	171302 (10.83)	31769 (12.33)	60052 (9.62)	37404 (9.29)	3088 (12.72)
500–999	595055 (29.41)	209378 (30.1)	43474 (31.85)	830437 (26.92)	424266 (26.83)	74772 (29.03)	159188 (25.51)	100016 (24.85)	6763 (27.85)
<500	1191732 (58.91)	398175 (57.24)	74027 (54.24)	1740916 (56.44)	903275 (57.12)	135597 (52.65)	360912 (57.84)	237694 (59.06)	11776 (48.5)
BMI (kg/m^2)	21.4 (19.7–22.1)	26.4 (24.7–28.4)	26.0 (24.4–28.0)	22.6 (21.1–24.1)	26.0 (24.5–27.8)	25.5 (23.9–27.1)	22.6 (20.9–24.2)	26.1 (24.5–27.8)	25.0 (23.4–26.7)
Systolic BP (mmHg)	115 (110-–121)	125 (118–131)	129 (120–135)	120 (110–130)	128 (119–135)	130 (120–138)	130 (119–138)	130 (120–144)	132 (123–142)
Diastolic BP (mmHg)	70 (67–80)	80 (70–84)	80 (75–85)	75 (70–80)	80 (72–85)	80 (76–89)	79 (70–82)	80 (71–86)	80 (74–88)
FBS (mg/dL)	88 (81–95)	93 (85–101)	94 (86–103)	92 (85–100)	98 (89–110)	100 (91–113)	95 (87–105)	100 (91–115)	103 (93–119)

Abbreviation: MASLD, metabolic dysfunction associated steatotic liver disease; ALD, alcohol-associated liver disease; MET, metabolic equivalent task; BMI, body mass index; BP, blood pressure; FBS, fasting blood sugar. Values are expressed N (%) and median (interquartile range), as appropriate.

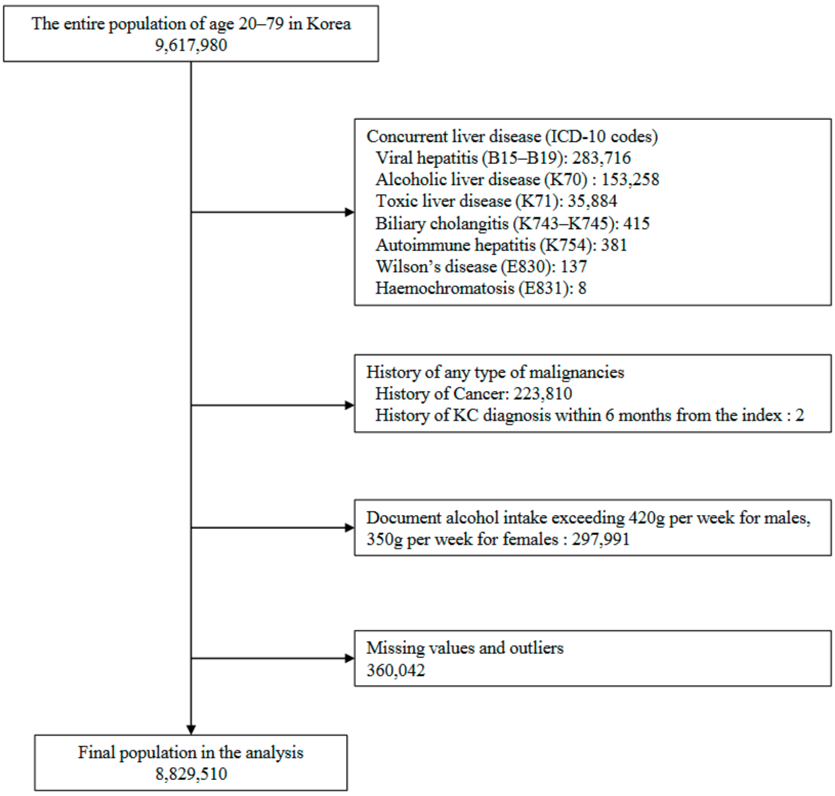

Figure S1. A heatmap for the risk of kidney cancer associated with the combination of fatty liver disease, alcohol intake, and the number of metabolic components.
